# Increased Zn/Glutathione Levels and Higher Superoxide Dismutase-1 Activity as Biomarkers of Oxidative Stress in Women with Long-Term Dental Amalgam Fillings: Correlation between Mercury/Aluminium Levels (in Hair) and Antioxidant Systems in Plasma

**DOI:** 10.1371/journal.pone.0126339

**Published:** 2015-06-15

**Authors:** María Eugenia Cabaña-Muñoz, José María Parmigiani-Izquierdo, Luis Alberto Bravo-González, Hee-Moon Kyung, José Joaquín Merino

**Affiliations:** 1 Centro CIROM, Centro de Implantología y Rehabilitación Oral Multidisciplinaria, Murcia, Spain; 2 Facultad de Medicina, Universidad de Murcia, UMU, Unidad Docente de Ortodoncia, Murcia, Spain; 3 Department of Orthodontics, Dental School, Kyungpook Nacional University, Daegu, Korea; 4 IUIN, Instituto de Investigación Neuroquímica, Universidad Complutense de Madrid, (U.C.M), Madrid, Spain; University of Sassari, ITALY

## Abstract

**Background:**

The induction of oxidative stress by Hg can affect antioxidant enzymes. However, epidemiological studies have failed to establish clear association between dental fillings presence and health problems.

**Objectives:**

To determine whether heavy metals (in hair), antioxidant enzymes (SOD-1) and glutathione levels could be affected by the chronic presence of heavy metals in women who had dental amalgam fillings.

**Materials and Methods:**

55 hair samples (42 females with amalgam fillings and 13 female control subjects) were obtained. All subjects (mean age 44 years) who had dental amalgam filling for more than 10 years (average 15 years). Certain metals were quantified by ICP-MS (Mass Spectrophotometry) in hair (μg/g: Al, Hg, Ba, Ag, Sb, As, Be, Bi, Cd, Pb, Pt, Tl, Th, U, Ni, Sn, Ti) and SOD-1 and Glutathione (reduced form) levels in plasma. Data were compared with controls without amalgams, and analyzed to identify any significant relation between metals and the total number of amalgam fillings, comparing those with four or less (n = 27) with those with more than four (n = 15). As no significant differences were detected, the two groups were pooled (Amlgam; n = 42).

**Findings:**

Hg, Ag, Al and Ba were higher in the amalgam group but without significant differences for most of the heavy metals analyzed. Increased SOD-1 activity and glutathione levels (reduced form) were observed in the amalgam group. Aluminum (Al) correlated with glutathione levels while Hg levels correlated with SOD-1. The observed Al/glutathione and Hg/SOD-1 correlation could be adaptive responses against the chronic presence of mercury.

**Conclusions:**

Hg, Ag, Al and Ba levels increased in women who had dental amalgam fillings for long periods. Al correlated with glutathione, and Hg with SOD-1. SOD-1 may be a possible biomarker for assessing chronic Hg toxicity.

## Introduction

Richardson *et al*. (2011) estimate that over 180 million Americans carry a total of more than one billion restored teeth (based on 2001–2004 population statistics) **[1].** Mercury toxicity varies between different types of mercury and its organic forms are much more toxic than inorganic forms **[2,3]**. Although dental amalgam fillings seem to be safe, they are nevertheless a significant chronic contributor to mercury body burden **[4,5,6]**. Heavy metals and oligoelements can be detected in human urine, plasma or hair samples by inductively coupled plasma-mass spectrometry (ICP-MS) **[7].** However, many epidemiological studies have failed to establish clear association between amalgam fillings presence and health problem **[2]**. The induction of oxidative stress by Hg is caused in part by the interaction between Hg and antioxidant enzymes **[8].** One way that cells remove ROS is by producing proteins such as glutathione and metallothionein that bind to ROS and form more hydrophilic compounds that are easily excreted through Glutathione S-transferases **[9]**. Copper and zinc-containing superoxide dismutase, (Cu/Zn- cytoplasmic enzyme (SOD-1) metabolizes superoxide radicals to molecular oxygen and hydrogen peroxide, providing defense against oxygen toxicity **[9,10,11]**. For this reason, there is a need to establish whether the Hg from dental amalgam fillings can increase heavy metal release and affect glutathione or SOD-1 activity as compensatory mechanism in patients (women).

## Objectives

The present study set out to quantify a plethora of heavy metals levels (μg/g: Al, Hg, Ba, Ag, Sb, As, Be, Bi, Cd, Pb, Pt, Tl, Th, U, Ni, Sn, Ti) in the hair of woman who had dental amalgam fillings for at least 10 years (average 15 years), comparing the data obtained with women control subjects without amalgams.

Data were analyzed to find out if the presence of these heavy metal levels was dependent of the total number of dental fillings. For this purpose, subjects (women) were divided into two groups: those with four or fewer dental amalgam fillings (<4; n = 27) and those with more than four in their mouth (>4; n = 15); pooled:dental Amalgam filling group, n = 42 (Amalgam).

Reduced glutathione levels and SOD-activity were also analyzed as possible compensatory mechanisms in the body of these woman. Lastly, data were analyzed to discover whether glutathione/SOD-1 activity (plasma) and Zn correlated with levels of heavy metals in subjects’ hair.

## Materials and Methods

The present study has been approved by the Institutional Review Board from Murcia University (UM; Spain) and following the Declaration of Helsinki. All Subjects in the present study have been properly instructed and they consent to participate by signing the appropriate informed consent paperwork. In addition, all efforts have been made to protect patient privacy and anonymity. CIROM Center has been approved and certificated by AENOR Spain (Spain; CIROM CERTIFICATE for dentist services (CD-2014-001 number; ER-0569/2014 following UNE-EN ISO 9001: 2008 as well as UNE 179001–2001 Directive from Spain). This study was developed following STROBE guidelines. The women subjects included in this experimental study fulfilled the following criteria:

Inclusion criteria Women who had four or less dental amalgam fillings (<4) in their mouth (n = 27); women subjects with more than four dental fillings (>4) (n = 15). All had dental amalgam fillings for at least ten years (average 15 years). Control subjects (women) without dental amalgam fillings were also included in the study (n = 13). All the women were matched in a similar age range (average age: 44 years; total number of amalgam fillings: Amalgam, n = 42). Other criteria were the absence of psychiatric disease, no previous history of DMSA use, subjects untreated (women) with prescription chelators, or any current treatment for iron deficiency (anemia). The women had no history of liver or kidney disease.

### Exclusion criteria

Physically handicapped women with metabolic diseases or progressive neurological disorders (4th Edition, DSM IV) were excluded, as were those taking regular medication such as stimulants, anticonvulsants, and atypical antipsychotic drugs.

### Sample analysis

The total sample was made up of 42 women, 42 who had dental amalgam fillings for at least ten years (average 15 years) and 13 control subjects (women); all were age-matched (mean age = 44 years). The dental amalgam filling group was divided into two groups: subjects (women) with a total number of amalgams greater than four (>4) (n = 15) and subjects with four or fewer dental amalgam fillings (<4) (n = 27). As no significant relation was found between heavy metal presence and the total number of amalgams in their mouth, the two groups were pooled into a single group (Amalgam group; pooled, n = 42). Mercury (Hg) is an essential constituent of dental amalgam fillings (around 50% in weight), together with Ag (41% in weight of the alloy powder), Sn (31%) and Cu (28%), and Zn on some occasions. Hair samples close to the scalp were taken from all subjects (0.25 g from the occipital area) and tested to determine these heavy metal levels by inductively coupled plasma-mass spectrometry (ICP-MS) (Doctor’s Data, USA); Doctor’s Data is a pioneer laboratory in toxicological analysis of heavy metals with over 35 years experience and provides testing to healthcare practitioners. ICP-MS values for heavy metals were reported in μg/g of hair. These patients (women) had a medium/higher sociocultural status.

### SOD-1 activity

SOD-1 activity was measured by means of a modified protocol as published Tiwari V, Chopra K (2013; **[12]**. The assay system consisted of 0.1 mM EDTA, 50 mM sodium carbonate and 96 mM of nitro blue tetrazolium (NBT). In the plate, 470 μl of above mixture was added to 30 μl of plasma and the auto-oxidation of hydroxylamine was observed by adding 0.05 ml of hydroxylamine hydrochloride (pH 6.0). Finally, SOD-1 activity was measured by the change in optical density at 560 nm for 2 min at 30/60 s intervals and normalized as Optical Density (D.O) by protein.

### Glutathione determination

The concentration of reduced glutathione was determined following the procedure described by Hissin and Hilf (1976) **[13]**. Sodium phosphate (0.1 M) plus EDTA (5 mM) (pH 8.0) and H_3_PO_4_ (25%) were added to the samples. The plasma was centrifuged for 30 min. at 13.000 r.p.m and supernatants were removed. Reduced glutathione levels were determined as follows: 200 μl of 40 mM N-ethylmaleimide and 100 μl of ortho-phthalaldialdehyde were added and fluorescence was measured at 350 m excitation and 420 nm emission, after 15 min of incubation at room temperature (RT).

### Statistical analysis

Data were analyzed using SPSS software (V17.0) and G Power. Mean and standard deviation were estimated for age and heavy metal levels in study group subjects’ hair and in control subjects (women). The Bonferroni and t Student test (2 tails) were applied to determine possible significant differences between women who had dental amalgam fillings (patients with four or less than four fillings and women with more than four fillings in their mouths) vs controls. Non parametric tests were applied when there was no homogeneity of variance (Mann Withney/Kruskal Wallis). Differences were considered statistically significant when p<0.05 and considered highly significant when p<0.01.

## Results

**Fig 1:**
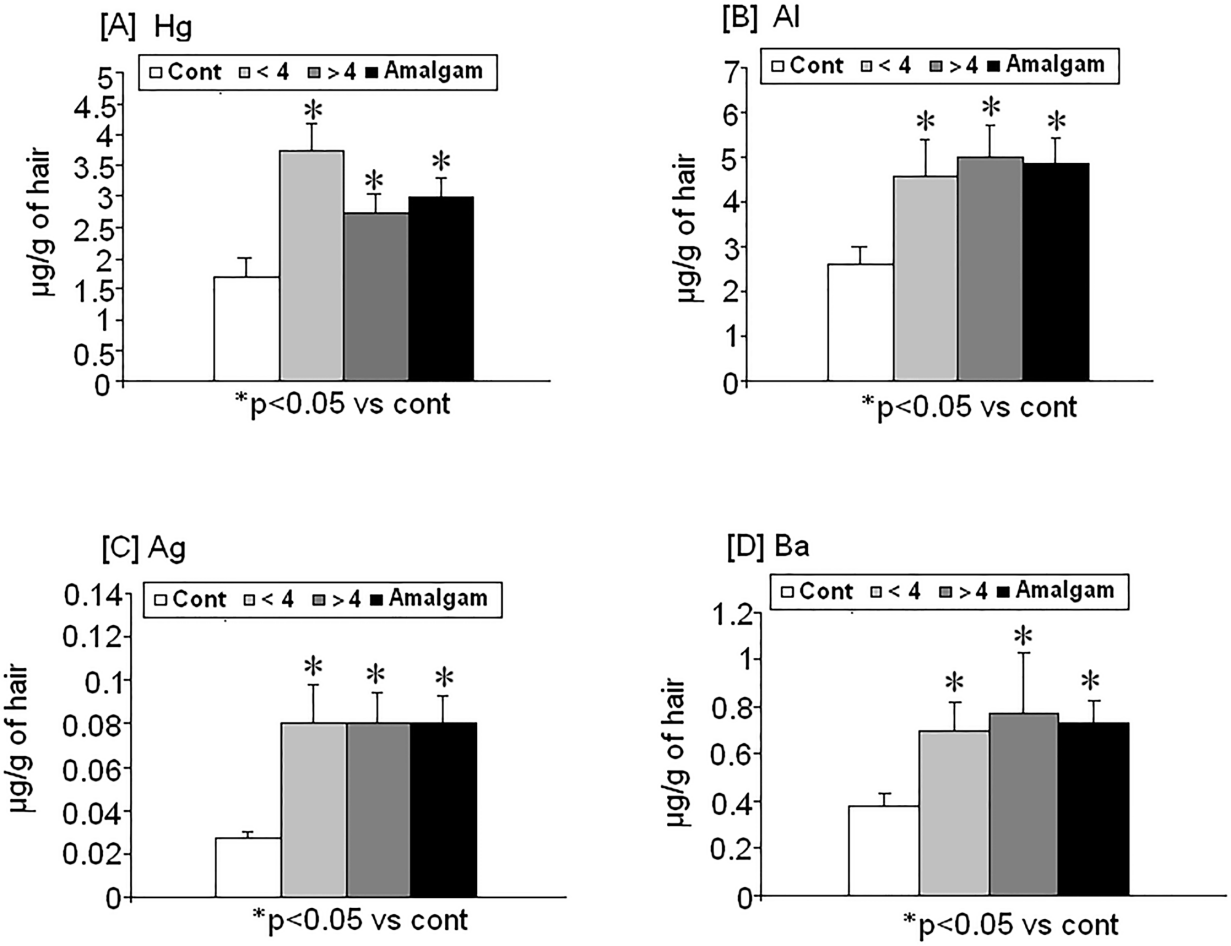
Changes in heavy metal levels in hair (μg/g of hair) from women with dental amalgam fillings for more than ten10 years (n = 42) as compared to controls (n = 13). [A] mercury (Hg), [B] aluminium (Al), [C] silver (Ag) and [D] barium (Ba). Amalgam (Pooled; n = 42); (<4; n = 27), (>4; n = 15), (control; n = 13). * p<0.05 vs control.

**There were** changes in heavy metal levels in hair from women with amalgam fillings for more than ten years**:** Hg, Al, Ag and Ba were higher in the hair of these subjects than in controls (Fig 1). Interestingly, data analysis identified a strong correlation between Hg and Al levels by in hair (r = 0,5; p<0.05; n = 42; r Pearson; data not shown)

### Hg levels

Hg was found to increase significantly in women with dental amalgams compared with control subjects without dental amalgams (p<0.05). However, both groups (<4 and >4) are not statistically different between them but they are significantly higher than controls (Fig 1A).

### Aluminum levels

Women with fillings (Amalgam group) showed higher Al levels than controls (p<0.029). Women with more than four (>4) fillings had increased Al levels as compared with control subjects (p<0.05) as did women with four or less (<4) (p<0.05). (Fig 1B)

### Silver levels

Women with dental amalgam fillings showed higher silver levels than controls (p<0.05) (Fig 1C)

### Barium levels

Women with less than four dental amalgam fillings (<4) and the total amalgam filling group showed higher Ba levels than the control subjects (p<0.05; Fig 1D).

The other heavy metals analyzed (Sn, Ni, Ti, Pb, Cd) were unaffected by the presence of dental amalgams in all cases (p>0.05; n,s). There were no differences for Sb, Be, Bi, Tl, Ti, U or either (data not shown) (Table 1)

**Table 1:** The analysis of the dental filling number and levels of other heavy metal levels in hair did not shown significant differences between both amalgam groups (total number of amalgams; Pooled; n = 42); >4 (n = 15) or <4 (n = 27), as compared to controls (n = 13).

(p>0.05) ns	Control	<4	>4	Amalgam
***Sn***	0.098 ± 0.025	0.69 ± 0.38	0.87 ± 0.57	0.75 ± 0.31
**Ni**	0.012 ± 0.025	0.0543 ± 0.038	0.51 ± 0.18	0.2 ± 0.067
***Ti***	0.446 ± 0.047	0.49 ± 0.071	0.394 ± 0.036	0.45 ± 0.047
***Pb***	0.93 ± 0.26	1.08 ± 0.2	0.48 ± 0.17	0.89 ± 0.14
***Cd***	0.013 ± 0.003	0.017 ± 0.005	0.19 ± 0.012	0.67 ± 0.0032

There were no differences for Sn, Ni, Bi, Ti. Pb, Cd or either (data not shown: Sb, Be, Bi, Tl, Th, U; μg/g).p>0.05: n.s (**Sb, Be, Bi, TI, Th, U**); (p>0.05; n.s; data not shown

**Fig 2:**
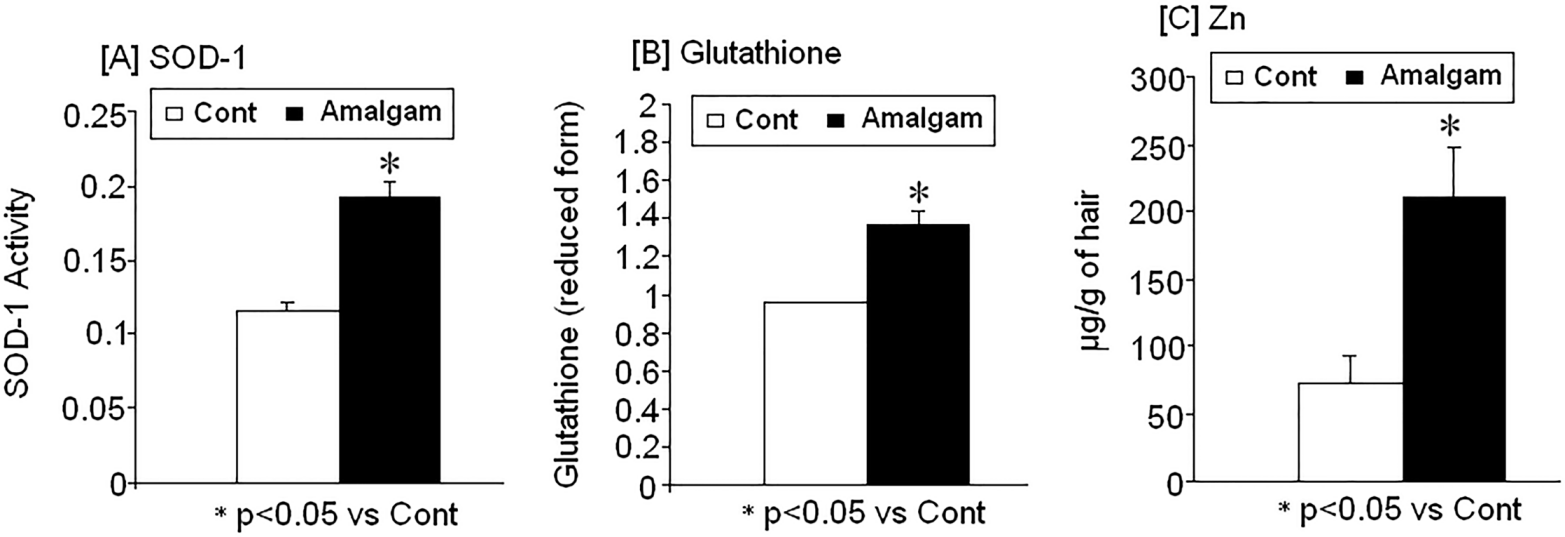
Changes on [A] superoxide dismutase activity SOD-1, [B] reduced glutathione and [C] Zn levels in women with dental amalgam fillings (n = 42) as compare to controls (n = 13). * p<0.05 vs control.

There were changes in SOD-1 activity, glutathione (reduced form) and Zn levels in women with long-term dental amalgam fillings: correlation between Hg/Al levels and antioxidant markers (SOD-1/Glutathione) (Fig 2)

#### Increased SOD-1 activity and glutathione

SOD-1 activity (Fig 2A) and glutathione (reduced form) (Fig 2B) increased in women with dental amalgam fillings compared with control subjects (p<0.05). Interestingly, there was a strong correlation between SOD-1 activity and the presence of Hg (r = 0.6; p<0.05), while Al levels were correlated with glutathione (r = 0.48, p<0.05; data not shown).

#### Zn

Zn was found to be significantly higher in women with dental amalgam fillings compared to control subjects without fillings (p<0.05; Fig 2C).

## Discussion

### Changes in levels of heavy metals in women with long-term dental amalgam fillings

Dental amalgams are the main source of the total mercury body burden in humans. This study found that mercury (Hg), silver (Ag), aluminum (Al) and barium (Ba) levels increased in the hair of women who had dental amalgam fillings more than ten years (average 15 years) in comparison with women control subjects without amalgam fillings (p<0.05). The values obtained agree with other studies that have reported 2–12 times higher Hg values in the body tissues of patients with dental amalgam fillings **[14,15]**. Ba levels were also seen to increase in women with dental fillings; this feature reflects the findings of another study in which increased Barium (Ba) levels exceeding the local permissible limits were found among dentists **[16]**. In the present study, Hg and Al levels were correlated between subject with fillings (r = 0.65; p<0.05), although Hg, Ag, Al, or Ba levels did not differ significantly between women with or more than four dental fillings in their mouths. These findings suggest that Hg could migrate from the oral cavity **[17]**. Moreover, other authors have also reported release of mercury from dental amalgam fillings **[18]** in agreement with the higher Hg levels observed in our study **[18]**. A recent study has analyzed levels of heavy metals (Hg, Sn) in dental pulp and blood samples from subjects with long-term amalgam restorations **[5]**. The study found heavy metal levels in blood and pulp blood samples in consonance with the higher Hg levels found in our study in hair. Moreover, other authors have also reported the release of mercury from dental amalgam fillings **[5,18]** in agreement with the higher Hg levels (hair) observed in our study. A clinical trial (England Children’s Amalgam Trial) of 534 children with fillings for more than five years reported higher mercury levels in agreement with the present study in women, **[6]**. In the study by Saghiri *et al*. (2014), the long-term presence of dental amalgam fillings (five years) did not induce remarkable changes in mercury levels within dental pulp tissue **[5]**. However, mercury levels increased in blood circulation five years after the placement of the restoration, which agrees with the highest Hg levels found (hair) in women with amalgam fillings in our study. Any discrepancies in Hg levels between different tissues (pulp, urine and hair) can be explained by the different periods the fillings considered in both studies, five years in the Saghiri *et al* (2014) study compared with at least ten years in the present study **[5]**. We applied an inclusion criterion of a minimum ten-year presence of dental amalgam fillings in the mouth because chronic exposure to various forms of mercury generated by the amalgam filling is characterized by a very long-lasting latency period of five to seven years before symptoms occur **[3].** In addition, women were divided according to the number of fillings: four or less or more than four dental amalgam fillings in their mouths [**19,20].** Although factors such as the number of years in the mouth, filling size and surface area contribute to the chronic mercury body burden [**17, 21]**, heavy metals levels were not different between women with four dental amalgam filling as compared to patients with more than four.

### Increased glutathione and SOD-1 activity as biomarkers of oxidative stress in women with dental amalgam fillings for more than ten years

Mercury levels in whole blood and urine are believed to be a reliable marker of recent exposure to inorganic and elemental mercury (Hg_0_). Piggato *et al*. 2013 reported that monitoring blood and urine is a useful means of identifying individuals with acute exposure to mercury but that the method might underestimate mercury retention and toxicity in tissues and organs [**22,21]**. For this reason, the present study measured Hg levels in hair, since mercury in blood and urine do not fully reflect the actual mercury burden in the human body [**21,14,15]**. Hg can affect antioxidant enzyme [23 **]**, like SOD-1 and catalase **[24]**. In fact, Hg produces reactive oxygen species (ROS), especially hydrogen peroxide (H_2_0_2_) and superoxide anion radicals (O2•), which are removed by SOD enzymes (Cu/Zn; SOD-1 and Mn; SOD-2) [**8,23]**. As methylmercury (MeHg) affects SOD-1 [**23]**, the present study set out to determine whether SOD-1 might be increased among women with amalgam fillings (with a mean age of 44 years). These subjects showed higher increases in glutathione levels than controls, suggesting that Hg can affect oxidative stress by interfering with antioxidant enzyme activities in women [**8,23,24]**. In our study, a positive correlation between Hg levels and oxidative stress-related SOD-1 activity was also observed (r = 0.6; p<0.05; n = 42), while Al was correlated with levels of glutathione (reduced form) (r = 0.48, p<0.05; n = 42), a finding that agrees with another study in which increased glutathione transferase (GPX3 and GSTM3) expression was observed in the blood of female double-crested Cormorants [**25]**. Since increased antioxidant activities have been seen to correlate to high mercury accumulations in the liver and kidneys [**24]**, it seems possible that higher glutathione (reduced form) and increased SOD-1 activity might reflect compensatory mechanism to the chronic presence of mercury in women. The increased SOD-1 activity observed agrees with human studies of fish-eating Amazonian communities, which identified an association between fish consumption and other oxidative stress biomarkers (catalase, GSH in blood) **[26]**, similarly to the SOD-1 and glutathione levels found in our study. This evidence suggests that glutathione and SOD-1 overactivation are crucial endogenous responses to prevent possible Hg toxicity in people with dental amalgams. Interestingly, Ala16Val MnSOD polymorphism (SOD-2) has been reported in cells exposed to methylmercury *in vitro* [**27].** In addition, *Plecotus auritus* (Golden-eared Bat) had altered expression of other antioxidant systems (GSTM3 mRNA levels) associated to high Hg levels (blood) **[25].** These increased endogenous antioxidant responses shown by glutathione and SOD-1 upregulation in women are in consonance with studies reporting that SOD-1 acted against methylmercury or cadmium toxicity ¨in vitro¨ [**28,29].** *Larus crassirostris* (Black-tailed Gull) had high metal concentrations (Mn and Al) in association with changes of antioxidant systems (blood) **[30,31].**

Al accumulation can induce CNS alteration and lead to dementia although there were no apparent physical alterations in woman with higher Al levels (hair) in the present study **[32]**. Interestingly, lower SOD-1 activity has been detected in elderly pre-diabetic patients compared with healthy control subjects **[33].** Thus, the enhanced SOD-1 activity could help maintain a healthy oxidative balance, which would otherwise have been disrupted by mercury **[24]** and other heavy metals released by the dental fillings [**16]**. In addition, (Zn) also rose in people with dental amalgams and SOD-1 is a Cu/Zn dependent enzyme **[34].** This could be the reason for which we fail to detect any physical alteration in women with long-term dental amalgam fillings [**31].** Thus, SOD-1 could be a potential biomarker in subjects with dental amalgam fillings. In a retrospective clinical study of medical records (41 patients), Piggato *et al*. (2013) measured mercury levels in samples of blood (n = 19), urine (n = 19), saliva (n = 20), and scalp hair (n = 17) to investigate the association between mercury levels and cases of multiple chemical sensitivity (MCS) [**22]**, MCS (also termed idiopathic environmental intolerance-IEI-) is a chronic condition characterized by an exaggerated body response to toxicants. The values detected for mercury in scalp hair (women) are close to values detected by Pigatto et al 2013 (hair 2.2 ± 2.5 μg/g vs 2.9 μg/g: Dental amalgam group) in our study; Hg: 7.6±13.6 μg/L (plasma); Hg 1.9±2.5 μg/L (urine); Hg (saliva) = 38.1±52.1 μg/L) [**22]**.

Finally, recent re-analyses of clinical trials suggest that susceptibility to mercury toxicity differs among individuals and can be influenced by genetic factors underestimated in some clinical trials [**35,36,37]**. However, some of these revised clinical studies have used insufficient time-frames as well as flawed measures of exposure such as blood or urine levels. In addition, many of them, particularly earlier studies, underestimated the genetic susceptibilities involved in patients [**6,15]**. As far as these authors are aware, the present study has produced the first clinical evidence for higher SOD-1 activity in plasma among women with long-term dental amalgam fillings, who showed higher mercury levels in their hair than control subjects without fillings [**16, 38]**. However, data must be interpreted with caution given the limited sample size (n = 55); the authors hope to confirm the results in further clinical trials. Nevertheless, SOD-1 may prove to be a clinically useful marker to determinate heavy metal contamination in humans. In fact, even 30 years after ceasing mercury exposure, dental nurses still show significant adverse health effects [**38, 39]**.

## Conclusions

Mercury (Hg), Ag, Al and Ba levels were increased in women with dental amalgam for more than years. The observed Al/glutathione and Hg/SOD-1 correlation could be adaptive response against the chronic presence of mercury. The findings suggest that increased SOD-1, and Glutathione levels could be biomarkers for chronic heavy metal toxicity in women with long-term amalgam fillings.

## References

[1] Richardson GM, Wilson R, Allard D, Purtill C, Douma S, Gravière J. Mercury exposure and risks from dental amalgam in the US population, post-2000. Sci Total Environ. 2011; 409: 4257–4268 10.1016/j.scitotenv.2011.06.035 21782213

[2] Park JD, Zheng W. Human exposure and health effects of inorganic and elemental mercury. J Prev Med Public Health. 2012; 45: 344–52 10.3961/jpmph.2012.45.6.344 23230464 PMC3514464

[3] Bellinger DC, Trachtenberg F, Barregard L, Tavares M, Cernichiari E, Daniel D, et al uropsychological and renal effects of dental amalgam in children: a randomized clinical trial. Journal of the American Medical Association. 2006; 15: 1775–83 10.1001/jama.295.15.1775 16622139

[4] Makhija SK, Gordan VV, Gilbert GH, Litaker MS, Rindal DB, Pihlstrom DJ, et al Practitioner, patient and carious lesion characteristics associated with type of restorative material: findings from the Dental Practice-Based Research Network. JADA. 2011; 142: 622–632. 21628683 10.14219/jada.archive.2011.0244 PMC3107519

[5] Saghiri MA, Banava S, Sabzian MA, Gutmann JL, Asatourian A, Ramezani GH, et al orrelation between long-term in vivo amalgam restorations and the presence of heavy elements in the dental pulp. J Trace Elem Med Biol. 2014; 28:200–4 10.1016/j.jtemb.2014.01.008 24731778

[6] Homme KG, Kern JK, Haley BE, Geier DA, King PG, Sykes LK, et al New science challenges old notion that mercury dental amalgam is safe. Biometals. 2014; 27:19–24 10.1007/s10534-013-9700-9 24420334 PMC3905169

[7] Puchyr RF, Bass DA, Gajewski R, Calvin M, Marquardt W, Urek K, et al Preparation of hair for measurement of elements by inductively coupled plasma-mass spectrometry (ICP-MS). Biol Trace Elem Res. 1998; 62:167–82 9676881 10.1007/BF02783969

[8] Farina M, Aschner M, Rocha JBT. Oxidative stress in MeHg induced neurotoxicity. Toxicol Appl Pharmacol. 2011; 256: 405–17 10.1016/j.taap.2011.05.001 21601588 PMC3166649

[9] Dierickx PJ. In vitro interaction of organic mercury compounds with soluble glutathione S-transferases from rat liver. Pharmacol Res Commun. 1985; 17:489–500 4034630 10.1016/0031-6989(85)90084-0

[10] Valko M, Leibfritz D, Moncol J, Cronin MTD, Mazur M, Telser J. Free radicals and antioxidants in normal physiological functions and human disease. The International Journal of Biochemistry & Cell Biology. 2007; 1: 44–84 10.1016/j.biocel.2006.07.001 16978905

[11] Merino JJ, Roncero C, Oset-Gasque MJ, Naddaf A, González MP. Antioxidant and protective mechanisms against hypoxia and hypoglycaemia in cortical neurons in vitro. Int J Mol Sci. 2014; 15:2475–93 10.3390/ijms15022475 24526229 PMC3958863

[12] Tiwari V, Chopra K. Resveratrol abrogates alcohol-induced cognitive deficits by attenuating oxidative-nitrosative stress and inflammatory cascade in the adult rat brain. Neurochem Int. 2013; 62:861–9 10.1016/j.neuint.2013.02.012 23422878

[13] Hissin PJ, Hilf RA. Fluorometric method for determination of oxidized and reduced glutathione in tissues. Anal. Biochem. 1976; 74: 214–226 962076 10.1016/0003-2697(76)90326-2

[14] Mutter J, Naumann J, Sadaghian C, Walach H, Drasch G. Amalgam studies: disregarding basic principles of mercury toxicity. Int J Hyg Environ Health. 2004; 207: 391–397 15471104 10.1078/1438-4639-00305

[15] Mutter J. Is dental amalgam safe for humans?. The opinion of the scientific committee of the European Commission. J Occup Med Toxicol. 2011; 6:2 10.1186/1745-6673-6-2 21232090 PMC3025977

[16] Shraim A, Alsuhaimi A, Al-Thakafy JT. Dental clinics: a point pollution source, not only of mercury but also of other amalgam constituents. Chemosphere. 2011; 84:1133–9 10.1016/j.chemosphere.2011.04.034 21543103

[17] Soussa E, Shalaby Y, Maria AM, Maria OM. Evaluation of oral tissue response and blood levels of mercury released from dental amalgam in rats. Arch Oral Biol. 2013; 58: 981–8 10.1016/j.archoralbio.2013.03.012 23611063

[18] Mortazavi SM, Neghab M, Anoosheh SM, Bahaeddini N, Mortazavi G, Neghab P, et al High field MRI and mercury release from dental amalgam fillings. Int J Occup Environ Med. 2014; 5: 101–5 24748001 PMC7767616

[19] Babi D, Vasjari M, Celo V, Koroveshi M. Some results on Hg content in hair in different populations in Albania. Sci Total Environ. 2000; 259: 55–60 11032135 10.1016/s0048-9697(00)00549-0

[20] Okati N, Sari AE, Ghasempouri SM. Biol Hair mercury concentrations of lactating mothers and breastfed infants in Iran (fish consumption and mercury exposure). Trace Elem Res. 2012; 149: 155–62 10.1007/s12011-012-9424-7 22592844

[21] Barrett RD, Bishara SE, Quinn JK. Biodegradation of orthodontic appliances. Part I. Biodegradation of nickel and chromium in vitro. Am J Orthod Dentofacial Orthop. 1993; 103: 8–14 8422037 10.1016/0889-5406(93)70098-9

[22] Pigatto PD, Minoia C, Ronchi A, Brambilla L, Ferrucci SM, Spadari F, et al. Allergological and Toxicological aspects in a multiple chemical sensitivity cohort. Oxid Med Cell Longev. 2013; 356235. 10.1155/2013/356235 Epub 2013 Dec 3 PMC3866722 24367721

[23] Ariza ME, Bijur GN, Williams MV. Lead and mercury mutagenesis: rolof H_2_0_2_, superoxide dismutase, and xanthine oxidase. Environ. Mol. Mutagen. 1998; 31: 352–361 9654245

[24] Hussain S, Atkinson A, Thompson SJ, Khan AT. Accumulation of mercury and its effect on antioxidant enzymes in brain, liver, and kidneys of mice. J Environ Sci Health B. 1999; 34: 645–60 10390852 10.1080/03601239909373219

[25] Gibson LA, Lavoie RA, Bissegger S, Campbell LM, Langlois VS. A positive correlation between mercury and oxidative stress-related gene expression (GPX3 and GSTM3) measured in female Double-crested Cormorant blood. Ecotoxicology. 2014; 23:1004–14 10.1007/s10646-014-1243-5 24788667

[26] Grotto D, Valentini J, Fillion M, Passos CJ, Garcia SC, Mergler D, et al Mercury exposure and oxidative stress in communities of the Brazilian Amazon. Sci Total Environ. 2010; 408:806–1. 10.1016/j.scitotenv.2009.10.053 19914681

[27] Algarve TD, Barbisan F, Ribeiro EE, Duarte MM, Mânica-Cattani MF, Mostardeiro et al In vitro effects of Ala16Val manganese superoxide dismutase gene polymorphism on human white blood cells exposed to methylmercury. Genet Mol Res. 2013; 12:5134–44 10.4238/2013.October.29.7 24301773

[28] Naganuma A, Miura K, Tanaka-Kagawa T, Kitahara J, Seko Y, Toyoda H, et al Overexpression of manganese-superoxide dismutase prevents methylmercury toxicity in HeLa cells. Life Sci. 1998; 62: PL157–61 9519816 10.1016/s0024-3205(98)00037-x

[29] López E, Arce C, Oset-Gasque MJ, Cañadas S, González MP. Cadmium induces reactive oxygen species generation and lipid peroxidation in cortical neurons in culture. Free Radic Biol Med. 2006; 40:940–51 16540389 10.1016/j.freeradbiomed.2005.10.062

[30] Kim M, Park K, Park J, Kwak I.Heavy metal contamination and metallothionein mRNA in blood and feathers of black-tailed gulls (*Larus crassirostris*) from South Korea. Environ Monit Assess 2013; 185:2221–2230 10.1007/s10661-012-2703-0 22692717

[31] Jenko K, Karouna-Renier NK, Hoffman DJ. Gene expression, glutathione status, and indicators of hepatic oxidative stress in laughing gull (*Larus atricilla*) hatchlings exposed to methylmercury. Environ Toxicol Chem. 2012; 31:2588–2596 10.1002/etc.1985 22890840

[32] Marques RC, Bernardi JV, Dórea JG, de Fatima R Moreira M, Malm O. Perinatal multiple exposure to neurotoxic (lead, methylmercury, ethylmercury, and aluminum) substances and neurodevelopment at six and 24 months of age. Environ Pollut. 2014;187:130–5 10.1016/j.envpol.2014.01.004 24486466

[33] Dzięgielewska-Gęsiak S, Wysocka E, Michalak S, Nowakowska-Zajdel E, Kokot T, Muc-Wierzgoń M. Role of lipid peroxidation products, plasma total antioxidant status, and Cu-Zn superoxide dismutase activity as biomarkers of oxidative stress in elderly prediabetics. Oxid Med Cell Longev. 2014; 987303. 10.1155/2014/987303 Epub 2014 May 5 PMC4026982 24891926

[34] Nedd S, Redler RL, Proctor EA, Dokholyan NV, Alexandrova AN. Cu,Zn-superoxide dismutase without Zn Is Folded but Catalytically Inactive. J Mol Biol. 2014; 426: 4112–24 10.1016/j.jmb.2014.07.016 25083917 PMC4258539

[35] Geier DA, Carmody T, Kern JK, King PG, Geier MR. A significant relationship between mercury exposure from dental amalgams and urinary porphyrins: a further assessment of the Casa Pia Children’s. Biometals. 2011; 2: 215–24 10.1007/s10534-010-9387-0 21053054

[36] Woods JS, Heyer NJ, Russo JE, Martin MD, Pillai PB, Farin FM. Modification of neurobehavioral effects of mercury by genetic polymorphisms of metallothionein in children. Neurotoxicol Teratol. 2013; 39: 36–4437. 10.1016/j.ntt.2013.06.004 23827881 PMC3795926

[37] Barcelos GR, Grotto D, de Marco KC, Valentini J, Lengert Av, de Oliveira AÁ, et al Polymorphisms in glutathione-related genes modify mercury concentrations and antioxidant status in subjects environmentally exposed to methylmercury. Sci Total Environ. 2013; 463–464: 319–325 10.1016/j.scitotenv.2013.06.029 23827356

[38] Khwaja MA, Abbasi MS. Mercury poisoning dentistry: high-level indoor air mercury contamination at selected dental sites. Rev Environ Health. 2014; 29:29–31 10.1515/reveh-2014-0010 24552960

[39] Jones L, Bunnell J, Stillman J. A 30-year follow-up of residual effects on New Zealand School Dental Nurses, from occupational mercury exposure. Hum Exp Toxicol. 2007; 26: 367–374 17615119 10.1177/0960327107076824

